# Correlations Between Hospitals’ Social Media Presence and Reputation Score and Ranking: Cross-Sectional Analysis

**DOI:** 10.2196/jmir.9713

**Published:** 2018-11-08

**Authors:** Justin D Triemstra, Rachel Stork Poeppelman, Vineet M Arora

**Affiliations:** 1 Department of Pediatrics Helen DeVos Children's Hospital Michigan State University College of Human Medicine Grand Rapids, MI United States; 2 Department of Pediatrics Nationwide Children's Hospital The Ohio State University College of Medicine Columbus, OH United States; 3 Department of Medicine Section of General Internal Medicine University of Chicago Chicago, IL United States

**Keywords:** social media, hospitals, benchmarking, hospital ranking

## Abstract

**Background:**

The US News and World Report reputation score correlates strongly with overall rank in adult and pediatric hospital rankings. Social media affects how information is disseminated to physicians and is used by hospitals as a marketing tool to recruit patients. It is unclear whether the reputation score for adult and children’s hospitals relates to social media presence.

**Objective:**

The objective of our study was to analyze the association between a hospital’s social media metrics and the US News 2017-2018 Best Hospital Rankings for adult and children’s hospitals.

**Methods:**

We conducted a cross-sectional analysis of the reputation score, total score, and social media metrics (Twitter, Facebook, and Instagram) of hospitals who received at least one subspecialty ranking in the 2017-2018 US News publicly available annual rankings. Regression analysis was employed to analyze the partial correlation coefficients between social media metrics and a hospital’s total points (ie, rank) and reputation score for both adult and children’s hospitals while controlling for the bed size and time on Twitter.

**Results:**

We observed significant correlations for children’s hospitals’ reputation score and total points with the number of Twitter followers (total points: *r*=.465, *P*<.001; reputation: *r*=.524, *P*<.001) and Facebook followers (total points: *r*=.392, *P*=.002; reputation: *r*=.518, *P*<.001). Significant correlations for the adult hospitals reputation score were found with the number of Twitter followers (*r*=.848, *P*<.001), number of tweets (*r*=.535, *P*<.001), Klout score (*r*=.242, *P*=.02), and Facebook followers (*r*=.743, *P*<.001). In addition, significant correlations for adult hospitals total points were found with Twitter followers (*r*=.548, *P*<.001), number of tweets (*r*=.358, *P*<.001), Klout score (*r*=.203, *P*=.05), Facebook followers (*r*=.500, *P*<.001), and Instagram followers (*r*=.692, *P*<.001).

**Conclusions:**

A statistically significant correlation exists between multiple social media metrics and both a hospital’s reputation score and total points (ie, overall rank). This association may indicate that a hospital’s reputation may be influenced by its social media presence or that the reputation or rank of a hospital drives social media followers.

## Introduction

The *US News and World Report* Best Hospital Rankings for adult and children’s hospitals have been released annually since 1990 [1,2]. These rankings affect the public reputation of a hospital and have been predicted to affect nonemergent volumes and total revenue by 5% in year-to-year changes in rankings [3]. Therefore, these rankings are highly anticipated by physicians and hospital administrators and are commonly used as marketing tools by hospitals to attract patients [4].

The final ranking of a hospital or subspecialty is determined by objective quality data in addition to subjective reputation data. The reputation score for each subspecialty is derived from annual surveys asking physicians where they would send their sickest patients, ignoring cost or location [1,2]. Owing to this subjective component of the total score, the *US News* hospital rankings have been criticized in recent years [5]. Therefore, *US News* has adjusted the composition of the final score, relying more on objective data in recent years [1,2]. However, Bush et al and Cua et al recently reported that the reputation of a hospital still disproportionately affects the final ranking in children’s [6] and adult hospitals by showing that the reputation component has a more significant influence on the total *US News* score than the objective components [7].

In this digital age, it is apparent that social media can influence how information is disseminated and the reputation of organizations; for example, *US News* uses a social media platform, Doximity, to distribute their annual survey to obtain the peer score for their annual rankings [2]. In addition, physicians in all fields have become increasingly engaged with social media [8]. Most hospitals have a Web-based social media presence [9] and use it for hospital promotion, education, community partnership, and fundraising purposes [10]. Therefore, because it has been shown that the reputation of a hospital contributes to *US News* rankings [6,7] and that social media presence can affect an organization’s reputation in today’s society, it would be important to understand whether social media presence correlates with *US News* hospital rankings. Interestingly, Ciprut et al found that the Twitter activity of urology departments was associated with *US News* rankings in Urology [11], although this analysis was not expanded to include other social media platforms or specialties. To address these gaps, this study aims to conduct a cross-sectional analysis of the adult and children’s hospitals’ social media presence over multiple platforms and a hospital’s *US News* rank to determine whether these correlations exist.

## Methods

### Hospital Ranking Data

We conducted a cross-sectional analysis of data from the 2017-2018 *US News and World Report* publicly available annual rankings for adult and children’s hospitals. *US News* hospital rankings are composed of both objective and subjective data that determine the total *US News* score; this score then determines the overall rank of the hospital within each subspecialty. The objective data are primarily based on survival outcomes, patient safety indices, and other care-related indicators, including nursing ratios, patient volume, and other subspecialty-specific quality of care data [1,2]. The subjective component consists of an annual survey of board-certified physicians who list up to 5 adult hospitals (up to 10 for children’s hospitals) that have the highest expertise in that subspecialty or where they would send their sickest patients, ignoring cost or location. This survey determines a hospital’s reputation score for that subspecialty [1,2]. Although the significance of the reputation component has been decreasing over time, it still composes 27.5% of the score for adult hospitals and 15% (8.5% in cardiology and heart surgery) for children’s hospitals.

Using these data, we determined a hospital’s total reputation score, as previously described in the literature, as the sum of each subspecialty reputation score for an individual hospital [6]. Although the total raw *US News* score is not reported for every hospital, the formula is reported to the public and was used to calculate each hospital’s total *US News* score [1,2]. These publicly available data were manually extracted from the 2017-2018 *US News* Adult and Children’s hospital reports. The descriptive statistics for these data can be viewed in those publicly available reports [1,2] with a summary of the statistics available in Multimedia Appendix 1. We used Microsoft Excel software (Microsoft Corporation) to store the data for this study.

### Statistical Analysis

The primary objective of this study was to analyze the association between social media metrics and *US News* rankings. Twitter, Facebook, and Instagram metrics for all hospitals that received a ranking in at least one subspecialty in *US News* were obtained on August 30, 2017, for children’s hospitals and October 12, 2017, for adult hospitals (Multimedia Appendix 1). For Twitter metrics, we obtained the number of followers, number of tweets, and Klout score (max=100), a metric that quantifies the Web-based social influence over the past 90 days, for each adult and children’s hospital with active accounts. All hospitals had active social media accounts for >90 days, ensuring that the Klout score represents an accurate estimation of the influence for all hospitals. Furthermore, the number of Facebook and Instagram followers were obtained for each adult and children’s hospital with an active account.

In this study, multiple regression analyses were used with social media metrics as the dependent variable. In one set of analyses, the independent variables were the total reputation score and the number of hospital beds at each institution. For the other set of analyses, the independent variables were the total points and the number of hospital beds at each institution. For analyses of tweets and Twitter followers, the number of months on Twitter was also included as one of the independent variables. Of note, we did not include a control for the active months of activity on Facebook and Instagram because they are not easily identifiable or reported. From these analyses, partial correlation coefficients were calculated between each social media metric and both the reputation score and total points. We defined statistical significance as *P*<.05. Analyses were performed using Stata v.15.0 (StataCorp).

## Results

### Children’s Hospitals

Table 1 summarizes the social media and correlation statistics for children’s hospitals when controlling for time on Twitter for Twitter metrics and bed size. Of the 79 children’s hospitals with at least one subspecialty ranked, 69, 68, and 46 hospitals had active Twitter, Facebook, or Instagram accounts, respectively. Children’s hospitals without an independent account were removed from the analysis. Twitter and Facebook followers correlated significantly with the total points (ie, higher rank) as well as with the reputation for children’s hospitals (*P*<.001).

**Table 1:** Social media metrics for US Children’s Hospitals and correlation with *US News* ranking and reputation score.

Social media metrics	Number of hospitals	Median (range)	Partial correlation coefficient for total points (*P* value)	Partial correlation coefficient for total reputation (*P* value)
Twitter followers	69	8306 (163-63,400)	.465 (<.001)	.524 (<.001)
Number of tweets	69	4714 (116-23,400)	.0181 (.93)	−.036 (.80)
Klout score	62	58 (23-81)	.117 (.38)	.067 (.61)
Facebook followers	68	59,141 (772-649,407)	.392 (.002)	.518 (<.001)
Instagram followers	46	4879 (260-5600)	.197 (.21)	.177 (.26)

### Adult Hospitals

Table 2 summarizes the social media and correlation statistics for adult hospitals when controlling for the bed size and time on Twitter for Twitter metrics. Of the 128 adult hospitals with, at least, one subspecialty ranked, 106, 105, and 62 had active Twitter, Facebook, or Instagram accounts, respectively. For adult hospitals, Twitter followers, the number of tweets, Klout score, Facebook followers, and Instagram followers correlated significantly with the total points (Table 2). Except for Instagram followers, these metrics also correlated significantly with the total reputation (Table 2). Hospitals with scores of 0 in the number of tweets and Klout score had active accounts with followers but no posts.

**Table 2:** Social media metrics for US Adult Hospitals and correlation with *US News* ranking and reputation score.

Social media metrics	Number of hospitals	Median (range)	Partial correlation coefficient for total points (*P* value)	Partial correlation coefficient for total reputation (*P* value)
Twitter followers	106	8074 (125-1,710,000)	.548 (<.001)	.848 (<.001)
Number of tweets	106	6087 (0-39,400)	.358 (<.001)	.535 (<.001)
Klout score	98	60 (0-92)	.203 (.05)	.242 (.02)
Facebook followers	105	18,582 (205-1,995,180)	.500 (<.001)	.743 (<.001)
Instagram followers	62	1615 (153-49,700)	.692 (<.001)	.831 (.26)

## Discussion

### Principal Findings

In this first study comparing social media metrics for adult and pediatric hospitals with *US News* rankings and reputation score, we found that both adult and children’s hospitals with more Twitter and Facebook followers had a higher 2017-2018 *US News* reputation score and total points (ie, overall rank). Although this correlation suggests that social media presence may influence the reputation of a hospital, it is also possible that the reputation and rank of a hospital drive social media followers. We also found that adult hospitals with more tweets and higher Klout scores, a measure of active social media participation, were associated with a higher reputation score and overall rank. Although it is possible that more prestigious hospitals have more followers because of prestige, it is also possible that this correlation may reflect that active engagement with social media may affect a hospital’s *US News* reputation score. Therefore, we have found marked correlations between a hospital’s social media metrics and its hospital rankings; however, further longitudinal studies will need to be conducted to determine causality.

### Limitations

There are a few limitations to our initial study. First, because social media metrics are cumulative, it is possible that hospitals which adopted social media earlier have larger followings and therefore, more posts. Furthermore, larger hospitals may have superior social media metrics because of increased resources for marketing and social media engagement. When controlling for time on Twitter and bed size of hospitals, these correlations remained significant, although other external factors, such as the size of the marketing department and total revenue of a hospital, may contribute as well. Second, owing to the cross-sectional analysis used in our study, we were unable to assess whether the increased social media presence over time correlates directly with an increase in reputation; this type of analysis could be conducted as a future study to confirm this finding. Third, because the subjective component of the rankings is based on physician voting, it would be helpful to know how many followers of a hospital are physicians; however, these data are not readily available, and usernames do not always identify the profession of their owner. Finally, Instagram correlated with both scores for adult and children’s hospitals, but it was only significant for the adult total score; this is likely because of Instagram being a newer platform with fewer hospitals active on Instagram.

### Conclusions

We believe that this study is the first to report the association between social media metrics and *US News and World Report* annual hospital rankings for both adult and children’s hospitals. This study establishes a correlation between increased social media engagement and *US News* rankings and reputation score for hospitals. Therefore, because previous studies have shown that physicians are increasingly engaged in large networks on social media [8] and *US News* rankings are disproportionately influenced by reputation scores [7], increasing a hospital’s social media presence could be a potential method of improving the reputation of hospitals and their rank in *US News* annual Best Hospital Rankings. Future studies should include a content analysis of Twitter, Facebook, and Instagram posts to determine the type of content being posted by hospitals and an assessment of the relationship between the use of social media and changes in hospital rankings over time to confirm whether increasing social media presence correlates with an increase in the hospital rank.

## Appendix

Multimedia Appendix 1 Summary of the total points, reputation score, bed size for adult and children’s hospitals, and descriptive statistics for each children’s hospital’s and adult hospital's social media data.
